# Plasma-derived exosome characterization reveals a distinct microRNA signature in long duration Type 1 diabetes

**DOI:** 10.1038/s41598-017-05787-y

**Published:** 2017-07-20

**Authors:** Marta Garcia-Contreras, Sanket H. Shah, Alejandro Tamayo, Paul D. Robbins, Ronald B. Golberg, Armando J. Mendez, Camillo Ricordi

**Affiliations:** 1 Diabetes Research Institute, University of Miami, Miami, FL USA; 2 School of Medicine and Dentistry, Catholic University of Valencia, Valencia, Spain; 3 Ri.Med Foundation, Palermo, Italy; 4 Sheila and David Fuente Graduate Program in Cancer Biology, University of Miami, Miami, FL USA; 5 The Scripps Research Institute, Jupiter, FL USA

## Abstract

Type 1 diabetes mellitus (T1DM) results from an autoimmune attack against the insulin-producing ß cells which leads to chronic hyperglycemia. Exosomes are lipid vesicles derived from cellular multivesicular bodies that are enriched in specific miRNAs, potentially providing a disease-specific diagnostic signature. To assess the value of exosome miRNAs as biomarkers for T1DM, miRNA expression in plasma-derived exosomes was measured. Nanoparticle tracking analysis and transmission electron microscopy confirmed the presence of plasma-derived exosomes (EXOs) isolated by differential centrifugation. Total RNA extracted from plasma-derived EXOs of 12 T1DM and 12 control subjects was hybridized onto Nanostring human v2 miRNA microarray array and expression data were analyzed on nSolver analysis software. We found 7 different miRNAs (1 up-regulated and 6 down-regulated), that were differentially expressed in T1DM. The selected candidate miRNAs were validated by qRT-PCR analysis of cohorts of 24 T1DM and 24 control subjects. Most of the deregulated miRNAs are involved in progression of T1DM. These findings highlight the potential of EXOs miRNA profiling in the diagnosis as well as new insights into the molecular mechanisms involved in T1DM.

## Introduction

Type 1 Diabetes Mellitus (T1DM), the most severe form of diabetes mellitus, is a disorder triggered by environmental factors that results in the autoimmune attack against the insulin-producing ß cells localized in the pancreatic islets of Langerhans. As a consequence, there is a decrease in insulin synthesis that leads to hyperglycemic episodes in T1DM subjects^1^.

Circulating autoantibodies against ß cell autoantigens are currently the only biomarkers clinically available^2^. However these autoantibodies don’t necessarily correlate with the loss of ß cells and are correlated with a relatively late stage of the autoimmune process and therefore are not suitable fo disease intervention^3^. To this end, there is a need for non-invasive biomarkers that will allow to early diagnose T1DM and to detect ß cell loss, whether this is caused by autoimmunity or other processes that can damage islet cells. Markers of ß cell loss may precede appreciable changes in insulin secretion, and thus may allow early intervention and prolong islet survival. Furthermore, biomarkers could be of assistance to determine the likelihood of development of islet autoimmunity. The expression of markers realted to autoimmunity in T1D have been shown to be maintained or even increased in long-duration cases^4^.

microRNAs (miRNAs) are small single-stranded non-coding RNAs of approximately 22 nucleotides in length, which function as posttranscriptional regulators of gene expression. Certain miRNAs have been shown to be specifically and highly expressed in pancreatic islets of langhergans^5, 6^ and thus are potential candidates for biomarkers of beta cell loss.

Exosomes (EXOs) are small (approximately 30–200 nm in diameter) lipid vesicles derived from multivesicular bodies that are released by virtually all cell types. EXOs have emerged as important mediators in cell communication, transferring proteins, lipids and RNA species (miRNA, mRNA, tRNAs, etc.) between cells^7, 8^. Moreover EXOs have been shown to be enriched in a subset of miRNAs found in the parental cell^9^. EXOs are present in different body fluids including serum, urine, cerebral spinal fluid, and saliva and broncheolar lavage fluid. The RNA content of EXO isolated from different sources can reflect biological events and disease processes^10–12^. In particular, plasma and serum exosome miRNA profiling have been shown to have a potential in the diagnosis of different diseases^11, 13^. Thus, plasma derived exosomes, enriched in specific miRNAs, could provide a disease-specific diagnostic signature allowing for prediction and monitoring of T1DM^9, 13^.

In this study, we measured the level of microRNAs in plasma-derived exosomes isolated by differential centrifugation from T1DM and control subjects, using nSolver microRNA microarray method. The significant differentially expressed miRNAs were then validated by qRT-PCR. The T1DM group exhibited an exosome miRNA profile distinct from the control subjects, demonstrating the feasibility of using a miRNA signature of plasma-derived exosomes as biomarkers of T1DM.

## Results

### Isolation and Characterization of exosomes

**Figure 1:**
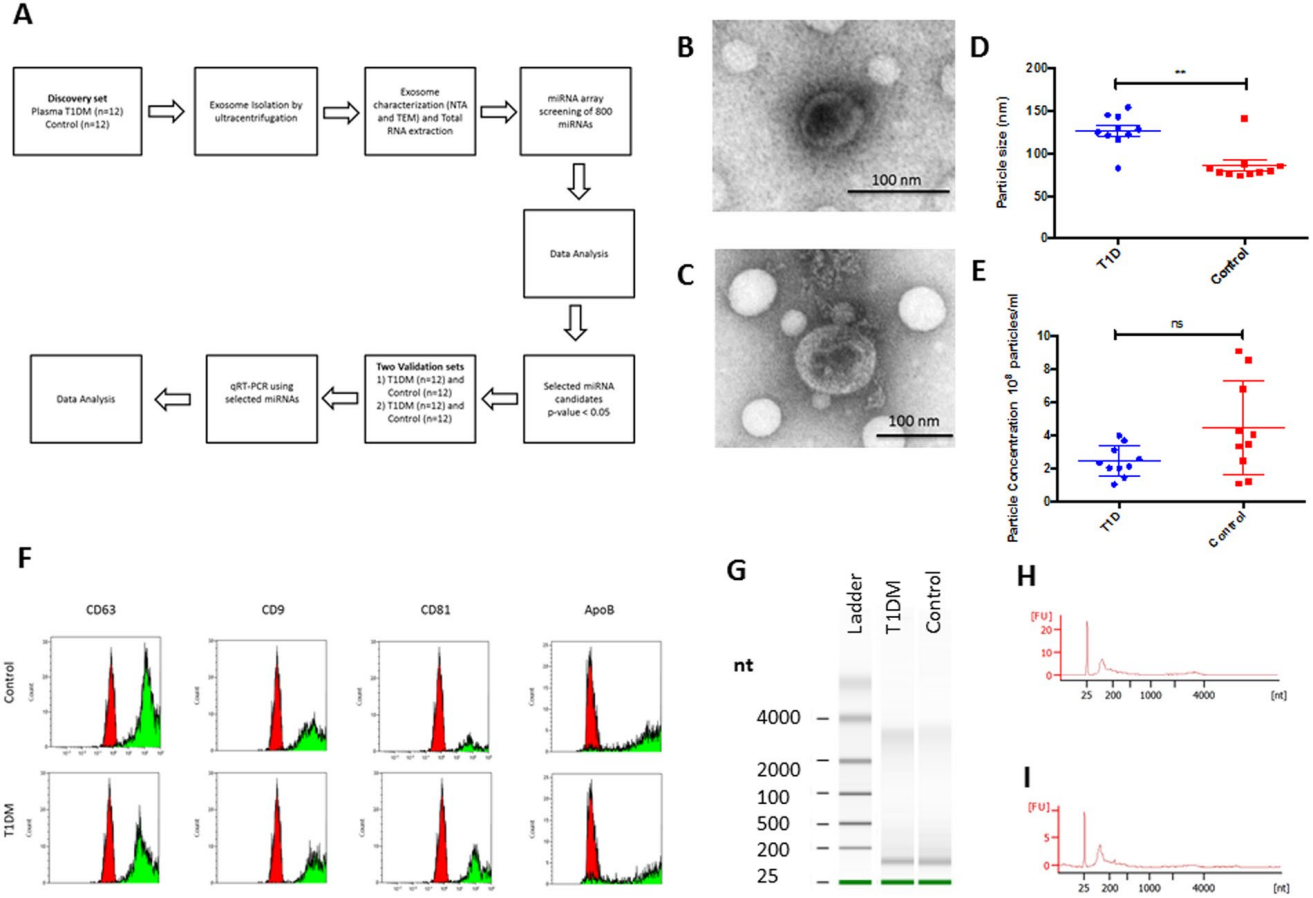
Work flow of study design and sample processing. Plasma from T1DM (n = 36) and control subjects (n = 36) was collected. Exosomes were isolated by ultracentrifugation and characterized by TEM and NTA analysis. Total exosome RNA was isolated and use for the miRNA microarray analysis (discovery set) or for the qRT-PCR validation (validation set) (**A**). Plasma exosomes were analyzed under electron microscopy which displayed the same morphology in T1DM and control subjects (**B**,**C**). Particle and Size distribution of exosomes analyzed by the Nanoparticle tracking analysis of T1DM and control subjects (**D**,**E**). Exosomal RNAs determined by the Agilent RNA Pico Chip. Exosomal RNA samples contained no detectable 18S and 28S rRNAs (**G**). Validation of selected exosome protein expression by flow cytometry (control read peak) (**F**). Small RNA Densitometry traces profiles were used to quantify and compare the relative abundance of various small RNAs in T1DM (**H**) and Control subjects (**I**).

Exosomes isolated from plasma of control subjects and T1DM subjects were analyzed by Nanoparticle tracking analysis (NTA) and transmission electron microscopy. The plasma-derived exosomes were within the normal range for exosome size (30–200 nm in diameter), and expressed CD63, CD9 and CD81 exosomal markers, consistent with previously reported characteristics of exosomes, apolipoprotein B (ApoB) also was detectable (Fig. 1D–F), and exhibited the characteristic cup-shape morphology (Fig. 1B,C). At this time, it is not clear if the presence of ApoB is due to the contamination with lipoproteins or if ApoB may be a component of the exosomes as previously reported^14, 15^.

Total RNA derived from T1DM and control subjects isolated exosomes was analyzed using Agilent Bioanalyzer small RNA chips (Fig. 1G–I). For both sets of samples, T1DM and Control subjects, the small RNA profiles were similar. The majority of exosome RNA content was small (<200 nt), which indicates that exosomes are enriched in short RNAs. These results are consistent with the isolated extracellular vesicles as being predominantly exosomes.

### Plasma-derived exosomes miRNA profiling

**Figure 2:**
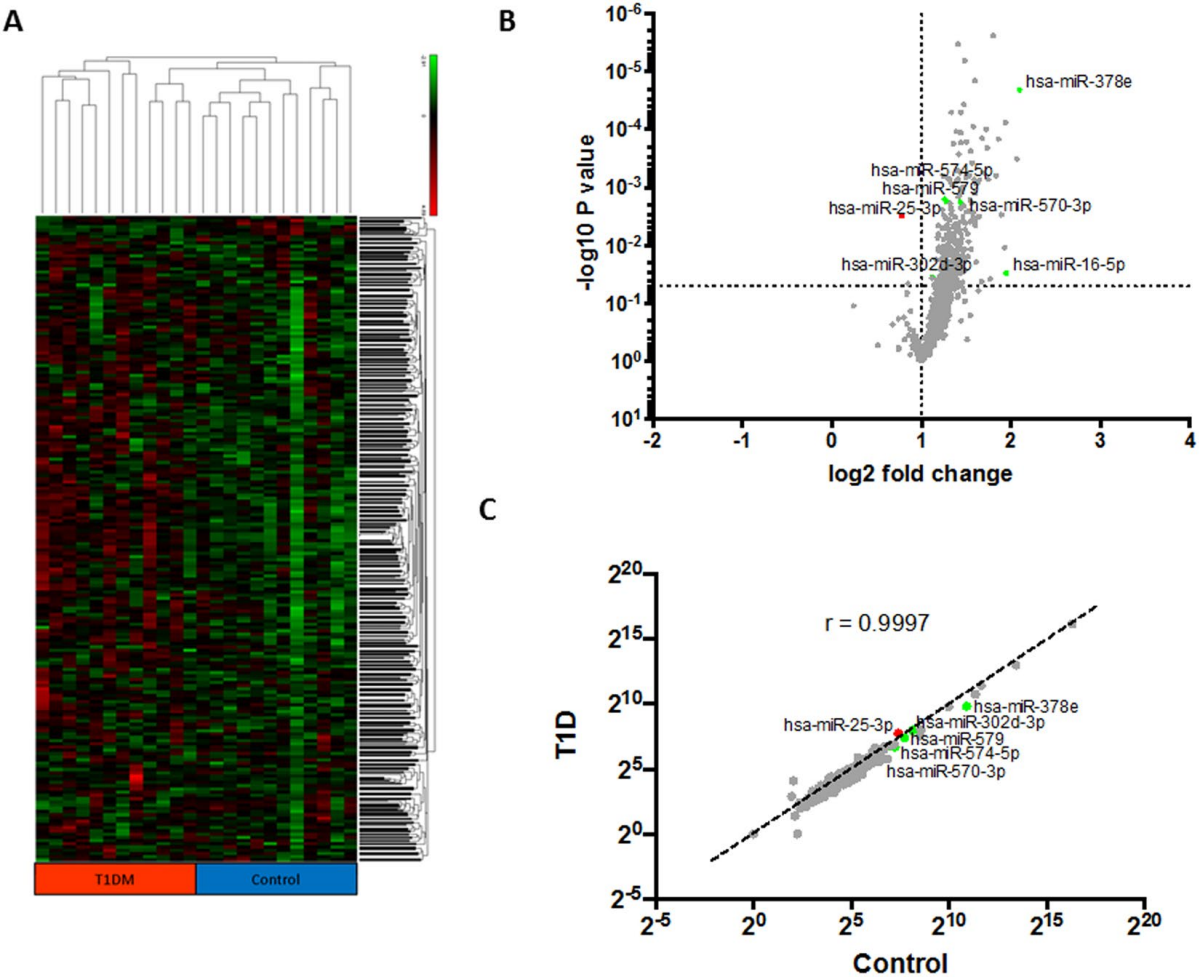
Differential miRNA content in T1DM and control subjects. Heatmap of the per-row normalized expression levels of selected miRNAs differentially expressed in T1DM and control subjects plasma-derived exosomes (**A**). Scatter plot of proteins enriched in Control or T1D subjects, n = 12 per group, using P < 0.05 (multiple t-test) and array threshold as cutoffs. Noted in the legend are Control subjects overexpressed miRNAs (green), T1D overexpressed miRNAs (red) (**B**). Correlation plot of exosome miRNAs, indicating in red and green those miRNAs expressed differentially T1DM and control subject’s exosomes (**C**).

**Figure 3:**
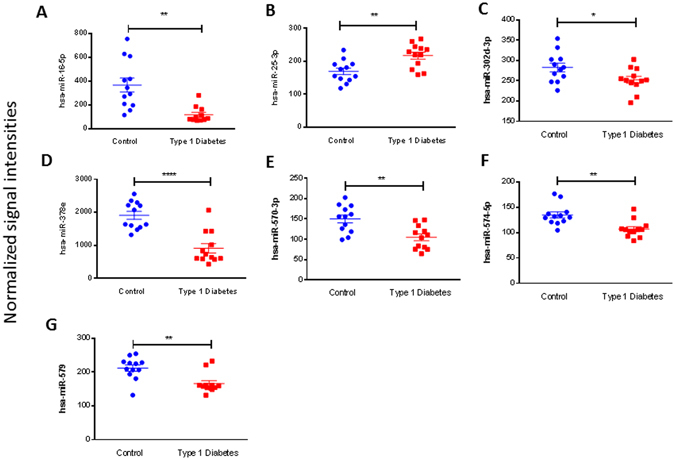
Differential microarray miRNA expression in T1DM and control subjects. Individual changes in the miRNA plasma exosome levels of the one up-regulated and six down-regulated miRNAs in T1DM patients (n = 12) and control subjects (n = 12). The signal intensities were normalized to the total signal intensity of the microarray. The horizontal lines indicate the mean normalized signal intensity for each group. Statistically significant differences were determined by Student’s t-test.

To screen for candidate plasma-derived exosome miRNAs associated with T1DM, miRNA microarrays were used to evaluate the two groups (T1DM and control) (Figs 2A and 3). As shown in the scatter plot (Fig. 2C), the microarray results identified various miRNAs that were differentially contained in the plasma exosomes of T1DM subjects samples relative to control subjects. Subsequently, we conducted pairwise comparison of the results of the scatter plot charts and found 7 miRNAs with significantly different expression between T1DM and the control. Six of these miRNAs miR-16, miR-302d-3p, miR-378e, miR-570–3p, miR-574-5p, miR-579; were down-regulated and one was up-regulated miR-25-3p in T1DM plasma-derived exosome samples compared to the control (p value < 0.05) (Figs 2B and 3).

### Housekeeping microRNA selection

Based on the literature and the microarray data, let-7, miRNA-631 and RNU6 were selected for testing in a random cohort of 24 plasma samples as possible “housekeeping” miRNA that could be used as an internal control. Hsa-miR-631, but not Let-7 and RNU6, showed non-significant differences between the groups (data not shown). These findings suggested miR-631 was the best internal control to use for assessing changes in exosomal miRNA levels.

### Verification of array data by qRT-PCR

**Figure 4:**
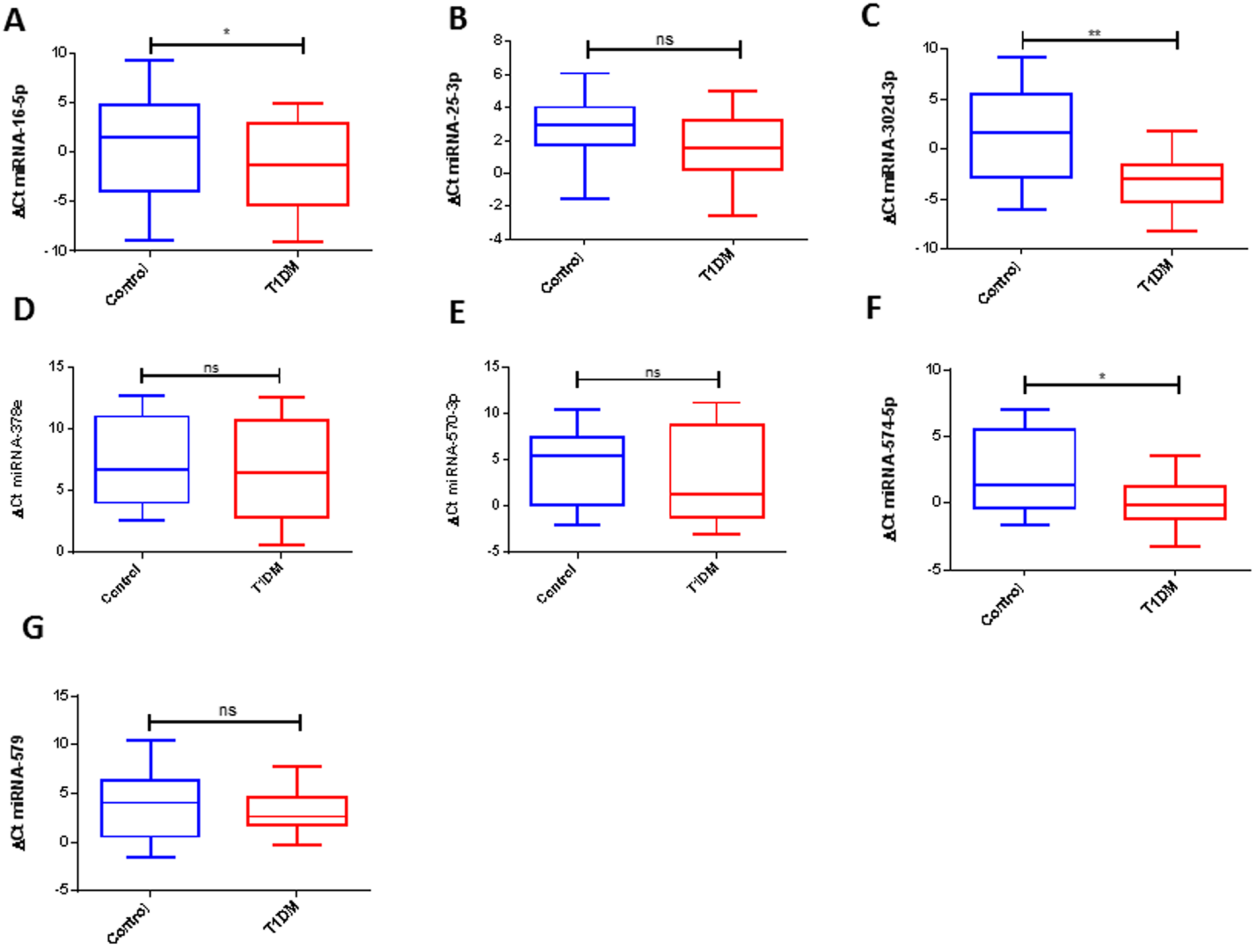
Box-and-whisker plots of the expression levels of the seven selected miRNAs in an independent set of T1DM (n = 24) and control subjects (n = 24). The comparative cycle threshold (Ct) method was used to quantify the levels of exosome miRNAs in T1DM and control subjects. The relative ratio was calculated using the ΔCt method. The Ct value of miR-631 was used as an internal standard. (*p-value < 0.05, **p-value < 0.01, ns = non significant).

To evaluate further the potential of these miRNAs as biomarkers for T1DM, the changes in the level of the individual miRNAs derived from the microarray dataset, were analyzed by real-time quantitative PCR in an independent cohort of plasma-derived exosomes from T1DM and control subjects. Interestingly, when examining the level of the miRNA in plasma exosomes in T1DM and control subjects, only miR-16-5p, miR-574-5p and miR-302d-3p were significantly higher in plasma exosomes from controls than those from T1DM (*p-value* < 0.05 and p-value < 0.01) (Fig. 4).

### Functional analyses of human islets co-incubated with plasma-derived exosomes

**Figure 5:**
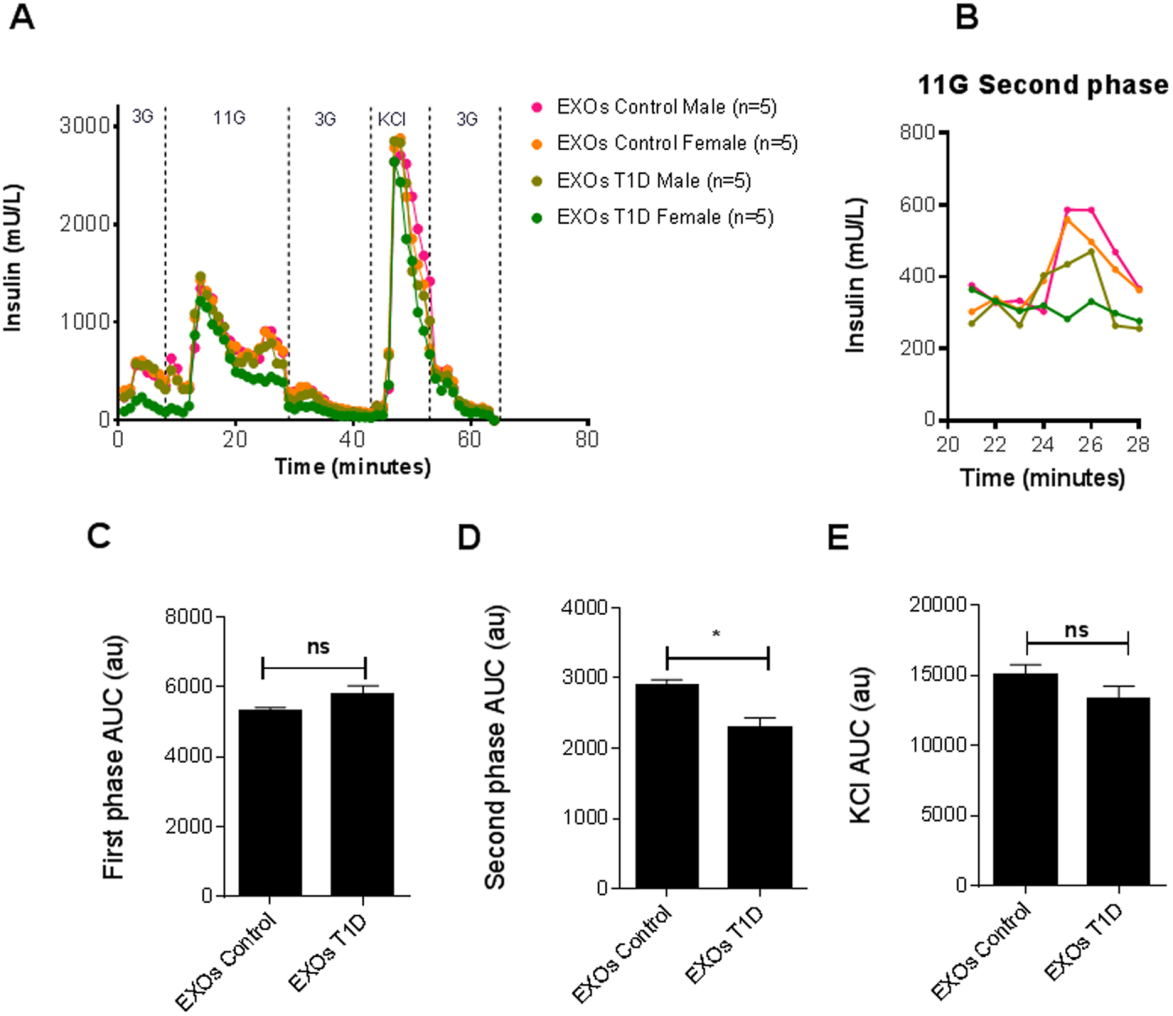
Effect of plasma-derived exosomes from T1D and controls on insulin secretion of perifused islets. Groups of 350 IEQ islets each, were stimulated every minute as shown in (**A**) for low (3 mM) → high (11 mM) → low (3 mM) glucose challenge followed by a KCl step. Pancreatic islet perifusion, normalized iInsulin secretion data to islet volume (IEQ). Data are expressed as ng insulin/islet equivalents (IEQs) per minute (**A**,**B**) or area under the curve (AUC) for each secretagogue (**C**–**E**). Statistical analysis was performed by Student’s *t*-test.

To demonstrate that exosomes from T1D patients are involved in the pathogenesis of T1D, we cultured isolated human islets in the presence of plasma-derived exosomes of T1D and controls, and determined the changes on the glucose stimulation (Fig. 5). The 11 mM glucose stimulation showed a similar biphasic pattern of insulin output, in T1D and control exosomes pre-treated samples; however, we observed a selective decrease on the second phase of the response in the T1D exosome-pretreated islets (Fig. 5D). These results may be an indicative of the effect of T1D exosomes on islet functionality.

## Discussion

In this study, we demonstrate for the first time that the miRNA signature found in exosomes isolated from the plasma of T1DM subjects could serve as a potential diagnostic biomarker. Although previous studies suggested the utility of plasma miRNAs in T1DM to our knowledge this is the first report on the quantitative assessment of miRNAs in exosomes isolated from plasma of T1DM patients.

There is a lack of robust biomarkers in T1DM that allow early diagnosis and intervention that could prolong islet survival^3^. In the last years, miRNA exosomes have emerged as ideal candidate biomarkers because that are stable in plasma and they appear to be tissue specific^9, 16^. Ribonucleases present in blood can degrade RNA, in comparison with whole plasma exosomes could prevent RNAse from degrading miRNAs providing an alternative source for miRNA biomarkers^9^. Moreover, miRNAs are short regulatory RNAs that control protein-coding genes and their expression is tissue specific. MiRNA expression pattern is altered during the progression of several diseases such T1DM where islets release miRNAs in circulation that are altered in diabetes subjects. Also pancreatic beta cells shed exosomes that contain miRNAs^17, 18^, which shows the possibility of using exosomes as a potential sources of biomarkers to early diagnose, disease progression, and reaction of autoimmune process in T1DM.

This study for the first time obtains by ultracentrifugation exosomes from plasma of T1DM that may also include other types of microvesicles released from the cell surface. It should be noted that due to lack of a nomenclature distinguishing exosomes from microvesicles^19^ we choose to use the term exosome to described this exosome-like vesicles obtained by ultracentrifugation and filtration of plasma in our study. This study, also characterizes the presence of plasma-derived exosomes in T1DM with surface expression of CD63, CD9 and CD81 via flow analysis. One limitation of our analysis was the lack of a validated housekeeping miRNA for exosomes to accurately compare exosome miRNAs levels by qRT-PCR^20^. Two types of housekeeping miRNAs have been used to normalize miRNA levels: spike-in controls (experiments need to be performed with same amount of cDNA) and “housekeeping” miRNAs. We selected RNU6 and let-7 that have been widely used as references for the normalization of qRT-PCR data of target miRNAs and miR-631 according to the microarray data analysis. We used hsa-miR-631 as the internal control because it had the most consistent level in plasma exosomes between the two groups mean value.

In the comprehensive microarray analysis, seven biomarker candidate miRNAs were identified and further analyzed with qRT-PCR. From those, all followed the same tendency in both techniques but only 3 microRNAS, miR-16-5p, miRNA-302d-3p, miR-574-5p, shown to be significant by qRT-PCR. This could be related to the differences of sensitivity and efficiency of the techniques^21^. We identified and confirmed that of the seven miRNAs whose levels change in plasma exosomes from T1DM subjects, 6 which were lower in T1DM patients (miR-16, miR-302d-3p, miR-378e, miR-570-3p, miR-574-5p, and miR-579) and 1 upregulated (miR-25-3p) several appear to play important roles in diabetes. For example, the level of hsa-miR-25-3p was reported to be significantly increased in the serum of new onset T1DM subjects, where it may have a role in glycemic control^22, 23^. MiR-16-5p was down-regulated after long-term exposure to elevated insulin and glucose levels in myoblasts^24^. Hoekstra *et al*.^25^ reported that a decrease in miR-302 expression predispose the liver to insulin resistance. Interestingly, it has been shown that miR-378 targets insulin-like growth factor receptor^26^ and is involved in lipid metabolism^27^. Adiponectin expression is negative correlated with the expression of miR-378^28^. miR-574 has been found in T1DM subjects with autoantibodies^29^. At the same time, decreased plasma miR-574 expression has been linked to diabetic nephropathy and type 2 diabetes patients^30, 31^. Furthermore, miR-16, miR-570, miR-574 and miR-579 have been found in extracellular vesicles released from human pancreatic islets^18^. Nevertheless, other common type 1 diabetes biomarkers, such as miR-375 correlated to beta-cell death in new onset patients^32^, were not found as potential biomarkers. This could be explain due to the long-term duration of the disease in the study population.

Dynamic glucose stimulation assay showed a selective decreased on the second phase of the response in the T1D exosome-pretreated islets. Interestingly, in type 2 diabetes instead of a biphasic pattern, there is a shift to a monophasic insulin release phase pattern^33^. The second phase is important for insulin release kinetics. The results may suggest an impairment in insulin release in human islets pretreated with T1D exosomes further impairing beta cell function. Taken together, our results show that we have been able to identify several circulating miRNAs that are deregulated in plasma-derived exosomes from patients with T1DM. This study provides suggestive evidence for the role of exosome miRNAs as clinical applicable biomarkers in T1DM. Future studies should be directed at discerning the role of these individual miRNAs in T1DM onset and T1DM progression.

## Materials and Methods

### Ethics statement

This study was conducted according to the principles expressed in the Declaration of Helsinki. Patients with T1DM attending the Diabetes Clinic at the Diabetes Research Institute/University of Miami Miller School of Medicine were recruited. Exclusion criteria included age <18 years, pregnancy, chronic kidney disease, a recent cardiovascular event or other systemic disease, or fibrate or niacin therapy. Healthy, non-diabetic subjects were recruited as controls by advertisement. BMI, medications, and duration of disease were assessed during the first study visit. This research study protocol was approved by the committee of the Human Subjects Research Office of the University of Miami (Miami, FL). Written informed consent was obtained from all study participants before blood collection.

### Research subjects

Thirty-six type 1 diabetic subjects attending the Diabetes Clinic at the Diabetes Research Institute, University of Miami Miller School of Medicine were prospectively enrolled in this study (Table 1). Exclusion criteria included age of 18 years, pregnancy, or therapy with fibrate or niacin. Thirty-six, non-diabetic subjects were recruited as control subjects. They denied a history of diabetes or prediabetes or fasting glucose elevation.

**Table 1:** Characteristics of the T1DM and control subjects included in the study.

	T1DM	Control
Gender (M/F)	23/23	23/23
Age, y	46.1 ± 14.4	44.2 ± 10.8
BMI, kg/m^2^	27.1 ± 4.8	25.7 ± 3.4
HbA1c, % (mmol/mol)	7.7 ± 1.3	NA
Diabetes duration, y	25.3 ± 15.9	NA
Serum Creatinine, mg/dL (μmol/L)	0.77 ± 0.2	0.84 ± 0.2
Total cholesterol, mg/dL	169.6 ± 34.3	205 ± 32.3
HDL cholesterol, mg/dL (mmol/L)	70 ± 19.7	56.9 ± 17.7
LDL cholesterol, mg/dL (mmol/L)	84.6 ± 27.3	123.3 ± 28.1
Triglycerides, mg/dL (mmol/L)	74.9 ± 28.9	119 ± 74.4

### Human Blood collection and plasma processing

Human Blood samples, from control subjects and type 1 diabetes subjects, were obtained after an overnight fast. Blood samples were collected in EDTA blood tubes and centrifuged to separate the plasma fraction. Plasma samples were then stored at −80 °C until further processing.

### Study design

Of the total thirty-six type 1 diabetic subjects retrospectively collected plasma samples, twelve were randomly selected for the initial discovery phase (Control subjects N = 12, T1DM subjects N = 12). We used a new cohort (Control subjects N = 24, T1DM subjects N = 24) for our validation analysis. In the discovery phase, miRNAs extracted from 12 T1DM subjects plasma samples were used to quantify a total of 800 miRNAs by microRNA analysis. Expression of 7 candidate miRNAs that were differentially expressed in the T1DM patient samples, were confirmed by an independent qRT-PCR set of samples (Control subjects N = 24, T1DM patients N = 24).

### Isolation of Exosomes from plasma

Exosomes were isolated from approximately 800 μL of cryopreserved plasma by differential centrifugation. Briefly, the plasma was diluted in PBS (1:1) and centrifuged at 2,000 g for 30 minutes at 4 °C and at 12,000 rpm for 45 minutes at 4 °C. Supernatants were collected into ultracentrifuge tubes and centrifuged in a Beckman TLA 120.2 fixed-angled rotor Ultracentrifuge (Beckman Coulter) at 110,000 g for 2 hours at 4 °C. Pellets were resuspended in PBS and filtered through a 0.22-µm filter (Millipore, Billerica, MA) and centrifuged at 110,000 × g for 70 minutes at 4 °C. Exosome pellets were washed with PBS and centrifuged at 110,000 × g for 70 minutes at 4 °C and resuspended in 200 μL of PBS. Exosomes preparations were conserved at −80 °C for later use.

### Nanoparticle tracking analysis

The concentration and size distribution of the isolated exosomes was analyzed by Nanosight NS300 system (Malvern Instruments Company, Nanosight, and Malvern, United Kingdom). Briefly, exosome preparations were homogenized by vortexing followed by serial dilution of 1:1000 in sterile Phosphate saline buffer and analyzed by NanoSight NS300. Each sample analysis was conducted for 90 minutes. Data was analyzed by Nanosight NTA 2.3 Analytical Software (Malvern Instruments Company, Nanosight, and Malvern, United Kingdom) with the detection threshold optimized for each sample and screen gain at 10 to track as many particles as possible with minimal background. Polystyrene latex standards were analyzed to validate the operation of the instrumentation and a blank 0.2 μm-filtered 1x PBS was also run as a negative control. At least three analysis were done for each individual sample.

### Transmission electron microscopy

Purified exosomes were fixed with 2% paraformaldehyde. A 20 μl drop of the suspension was loaded onto a formvar coated grid, negatively stained with 2% aqueous uranyl acetate for 2 minutes, and examined under a transmission electron microscope FEI Tecnai G2 Spirit using a digital camera Morada (Olympus Soft Image Solutions).

### Flow cytometry analysis

Exosomes were analyzed for the presence of exosomal markers CD63, CD9, CD81 and ApoB by flow cytometry. Isolated exosomes by ultracentrifugation were resuspended in PBS and bound to magnetic CD63-coated Dynabeads (Life Technologies, Grand Island, NY, USA) during an overnight incubation at 4 °C. The following day the Dynabeads-bound exosomes were stained with the corresponding antibodies along with appropriate Isotype control (green peak), and analyzed by flow cytometry (Beckman coulture Cytoflex Flow Cytometer).

### Total RNA isolation

Exosome total RNA was prepared using a mirVana™ miRNA Isolation Kit (Life technologies, Carlsbad, California). The isolation procedure was followed according to the manufacturer’s standard protocol. The quantity and quality of the RNA were determined by their concentration and purity (A260/A280 and A260/A230) was recorded by using the NanoDrop ND1000 (NanoDrop Technologies, Waltham, MA, USA) and Agilent Bioanalyzer 2100 with a Small RNA Chip (Agilent Technologies, Santa Clara, CA, USA).

### miRNA microarray and NanoString miRNA expression analysis

Total exosome RNA was used for human NanoString nCounter miRNA microarray assay (Nanostring Technologies, Seattle, WA, USA) according to the manufacturer’s instructions. The nCounter Human miRNA Panel v2 that evaluates 800 miRNAs was used. miRNAs extracted from exosomes was subjected to nCounter miRNA sample preparation according to the manufacturer’s instructions. This was followed by ligation of 100 ng of miRNA and hybridization to probes at 65 °C for 18 hours following the manufacturer’s protocol. Next day, the hybridized probes were purified and counted on nCounter Prep Station and Digital Analyzer. The data obtained from Analyzer contained counts of individual fluorescent barcodes and thus a count of miRNAs present in the sample. The nCounter results were analyzed by the nSolver 2.0 software according to the manufacturer’s instructions (Nanostring Technologies, Seattle, WA, USA). Data are available in GEO Omnibus database accession number GSE97123.

### Quantitative real-time PCR assay

To validate the RNA sequencing data, we performed a qRT-PCR analysis of hsa-let-7, hsa-miR-631, RNU6, hsa-miR-16-5p, hsa-miR-25-3p, hsa-miR-302d-3p, hsa-miR-378e, hsa-miR-570-3p, hsa-miR-574-5p, and hsa-miR-579. Transcription and amplification followed routine procedure. Real-time PCR was performed using SYBR PCR kit (Qiagen), StepOne Real-Time PCR System, and statistical analysis by the ΔCt method according to the supplier’s recommendation (Applied Biosystems). Primers are listed in Table 2.

**Table 2:** Primer list.

miRNA	miRBase ID	sequence
hsa-miR-16-5p	MIMAT0000069	uagcagcacguaaauauuggcg
hsa-miR-25-3p	MIMAT0000081	cauugcacuugucucggucuga
hsa-miR-302d-3p	MIMAT0000718	uaagugcuuccauguuugagugu
hsa-miR-378e	MIMAT0018927	acuggacuuggagucagga
hsa-miR-570-3p	MIMAT0003235	cgaaaacagcaauuaccuuugc
hsa -miRNA- 574-5p	MIMAT0004795	ugagugugugugugugagugugu
hsa -miRNA-579	MIMAT0003244	uucauuugguauaaaccgcgauu
hsa -miRNA- 631	MIMAT0003300	agaccuggcccagaccucagc
hsa -miRNA-let-7	MIMAT0000062	ugagguaguagguuguauaguu
hsa-RNU6-2	—	acgcaaattcgtgaagcgtt

### Evaluation of Appropriate Housekeeping microRNA

Because of the absence of a validated reference genes in the plasma exosome samples for the normalization of exosome microRNA expression data, it was critical to choose an appropriate housekeeping microRNA. According to the literature let-7, RNU6 that have been widely used to normalize miRNAs in plasma and the miRNA data hsa-miR-631, were tested by qRT-PCR.

### Co-incubation of human plasma-derived exosomes with human Islets

Isolated human islets were plated at the cell concentration of 300 IEQ per well of six-well plates. Isolated exosomes (pooled from 5 male or female (isolated from 250ul plasma), control or T1D patients) were added to each well and the plate was incubated for 48 h at 37 °C.

### Dynamic Glucose-Stimulated Insulin Release (GSIR)

A subset of islets (n = 4) were also subjected to dynamic GSIR perifusion studies. GSIR perifusion experiments were performed as described before^34^ using a custom built apparatus that allows parallel perifusion in up to eight channels (PERI-04, Biorep, Inc., Miami, FL). Briefly, one hundred islets were handpicked and loaded in Perspex microcolumns, between two layers of acrylamide-based microbeads slurry (Bio-Gel P-4, Bio-Rad Laboratories, Hercules, CA). Perifusing buffer containing 125 mM NaCl, 5.9 mM KCl, 1.28 mM CaCl2, 1.2 mM MgCl2, 25 mM HEPES, 0.1% bovine serum albumin, and 3 mM glucose at 37 °C with selected glucose (low = 3 mM; high = 11 mM) or KCl (25 mM) concentrations was circulated through the columns at a rate of 100 μL/min. After 45–60 minutes of washing with the low glucose solution for stabilization, islets were stimulated with the following sequence: 5 min of low glucose, 20 min of high glucose, 15 min of low glucose, 10 min of KCl, and 10 min of low glucose. Serial samples (100 μL) were collected every minute from the outflow tubing of the columns in an automatic fraction collector designed for a multi-well plate format. The sample container harboring the islets and the perifusion solutions were kept at 37 °C in a built-in temperature controlled chamber. The perifusate in the collecting plate was kept at <4 °C to preserve the integrity of the analytes. Insulin concentrations were determined with a commercially available ELISA kit (Mercodia Inc., Winston Salem, NC). Values obtained with the human kit are in mU/L; they were converted to μg/L using using 1 µg/L = 23 mU/L per the guidelines of the manufacturer. To account for possible differences in viability/purity across experiments as well as in IEQ numbers among islets in different channels, values were rescaled by up to 30% taking into account the KCl-induced release as a normalization factor for each condition.

### Statistical Analysis

Data are expressed as means ± SEM. Two-tailed Student t tests were applied to assess differences. According to request, assays (three repetitions, regardless of intra-assay triplicates) were statistically evaluated by the corresponding statistical test. *P* values of <0.05 were considered significant.
